# Spatiotemporal Variation of Sediment Export from Multiple Taiwan Watersheds

**DOI:** 10.3390/ijerph16091610

**Published:** 2019-05-08

**Authors:** Li-Chi Chiang, Yung-Chieh Wang, Ci-Jyun Liao

**Affiliations:** 1 Department of Civil and Disaster Prevention Engineering, National United University, Miaoli City 36063, Taiwan; lchiang@nuu.edu.tw (L.-C.C.); qaz147e@yahoo.com.tw (C.-J.L.); 2 Department of Soil and Water Conservation, National Chung Hsing University, Taichung 402, Taiwan

**Keywords:** soil erosion, sediment transport, typhoons, watershed management

## Abstract

Soil erosion and landslide triggered by heavy rainfall are serious problems that have threatened water resources in Taiwan watersheds. This study investigated the relationship among streamflow, sediment load, sediment concentration and typhoon characteristics (path and rainfall amount) during 2000–2017 for nine gauging stations in five basins (Tamshui River basin, Zhuoshui River basin, Zengwen River basin, Gaoping River basin, and Hualien River basin) representing the diverse geomorphologic conditions in Taiwan. The results showed that streamflow and sediment load were positively correlated, and the correlation was improved when the sediment load data were grouped by sediment concentration. Among these basins, the Zhuoshui River basin has the highest unit-discharge sediment load and unit-area sediment load. The soil in the upstream was more erodible than the downstream soil during the normal discharge conditions, indicating its unique geological characteristics and how typhoons magnified sediment export. The spatiotemporal variation in sediment loads from different watersheds was further categorized by typhoons of different paths. Although typhoon path types matter, the Zhuoshui and Hualien River basin were usually impacted by typhoons of any path type. The results indicated that sediment concentration, the watershed soil characteristics, and typhoons paths were the key factors for sediment loads. This study can be useful for developing strategies of soil and water conservation implementation for sustainable watershed management.

## 1. Introduction

Located at the junction of the Philippine Sea Plate and the Eurasian Plate, the high terrain in Taiwan was formed due to the strong orogeny and frequent earthquakes. Taiwan has young geological property and fragile rock formation. Taiwan is the 13th highest precipitation country in the world, with an average annual rainfall of 2500 mm. The rapid flows from steep slopes are difficult to store in rivers. Due to the spatiotemporally uneven rainfall distribution and high population density, the available rainfall per person is less than one-sixth of the world’s average value, which indicates that Taiwan is the 18th high water-deficient country in the world [1]. Thus, reservoirs have been built in middle and upper reaches of rivers for water storage. In order to meet the increasing water demand from industry, agriculture, and municipal usage, water and soil conservation in upstream hillslopes are important to extend the reservoir life. Nevertheless, the balance of sediment transport in rivers can still be affected by land development in a basin. During the wet season, typhoons invade Taiwan with heavy rainfall that wash hillslopes, causing landslides and debris flow into downstream areas. Many of the sediment disasters are caused by serious scouring of siltation in rivers, such as large-scale constructions in upstream areas and channels, destruction of vegetation, and excessive sediment gravels that seriously influence the sediment balance in rivers. The overloaded sediment yields brought by stormflows frequently cause serious river bank damage and bed scours, which further threaten the lives and property of conservation structures.

Sediment yields in estuary areas are mainly from middle and upstream reaches, which can be traced back to sediments generated by slope failure, hillslope erosion and river bed scouring [2]. Materials in rivers can be transported by three pathway types, which are dissolved matter, suspended load and bed load. The dissolved matter refers to materials that dissolve in water as ions and move with the flow; the suspended load majorly consists of clays, silts and fine sands, which are transported in suspension; the bed load are usually large sands, gravels and cobbles, and move through saltation, rolling and sliding near the bed [3]. Because bed load is difficult to measure and the more easily-measured suspended load accounts to a significant portion of the total sediment load, most studies focus on the suspended load when dealing with the issues of sediment transport, e.g., [4,5,6,7,8]. The high positive dependency between the suspended load and discharge has been reported since larger discharge of faster and more turbulent flow has larger hydrodynamic forces to carry sediments [9,10,11]. A sediment rating curve is the correlation between the suspended sediment concentration and discharge. This method has been widely applied and the power function is the most frequently used among various calibrating equations [6,8,12,13,14,15]. However, such positive correlation shows distinctive differences among dates, seasons, and regions [11]. Thus, calibration of rating curves is necessary to investigate the suspended load-discharge correlations among different areas in different seasons. Based on this reason, the regional and seasonal variations of rating curves should gain their attention in the studies focusing on the sediment transport issues in Taiwan.

Rainfall variation can result in sediment disasters. Many studies have reported that the precipitation decreased in southern Taiwan and increased in northern Taiwan [16,17], and the number of annual rainy day has significantly decreased in response to climate change [18]. However, trends of increasing maximum daily rainfall and the average daily rainfall intensity of a year. This indicates that more precipitation has been received during a year but the precipitation was more concentrated to fewer days and caused more intensive rainfall events, resulting in a higher frequency of flood and more serious damage caused by relative disasters [18]. Some critical rainfall conditions, such as rainfall intensity, duration, and accumulative precipitation, were proposed as the thresholds for debris flow and/or landslide occurrence and warning announcement [19,20,21,22,23]. Besides the rainfall variation, extreme weather events (i.e., heavy rains and typhoons) can also affect sediment loads in rivers. The discharge during extreme events is recognized as the major transporting force of the suspended load [2,4,24]. Reference [25] showed that the major source of sediment discharge in Sinwulyu River basin in eastern Taiwan was brought by typhoon and heavy rainfall events by analyzing the satellite images for landslide distribution after six typhoons and two earthquakes occurred in that region. Moreover, Taiwanese studies have indicated that typhoon and heavy rain events have led to significant increase from tenfold to couple hundred times in the suspended load of rivers during the peak discharge in Shihmen Reservoir basin [26] and Zengwun River basin [27]. [5] proposed that during 1975–1996 suspended sediment loads during typhoon events accounted for 59% of the total suspended load but typhoon duration only accounted for 6.47% of the total duration. 

This study aims to investigate the correlation between the sediment load and discharge in Taiwan by studying the relationships among streamflow, sediment transport, sediment concentration, and maximum daily typhoon rainfall during 2000–2017 for nine gauges in five basins (Tamshui River basin, Zhuoshui River basin, Zengwun River Basin, Gaoping River basin, and Hualien River basin). 

## 2. Materials and Methods

### 2.1. Study Area and Data

**Figure 1:**
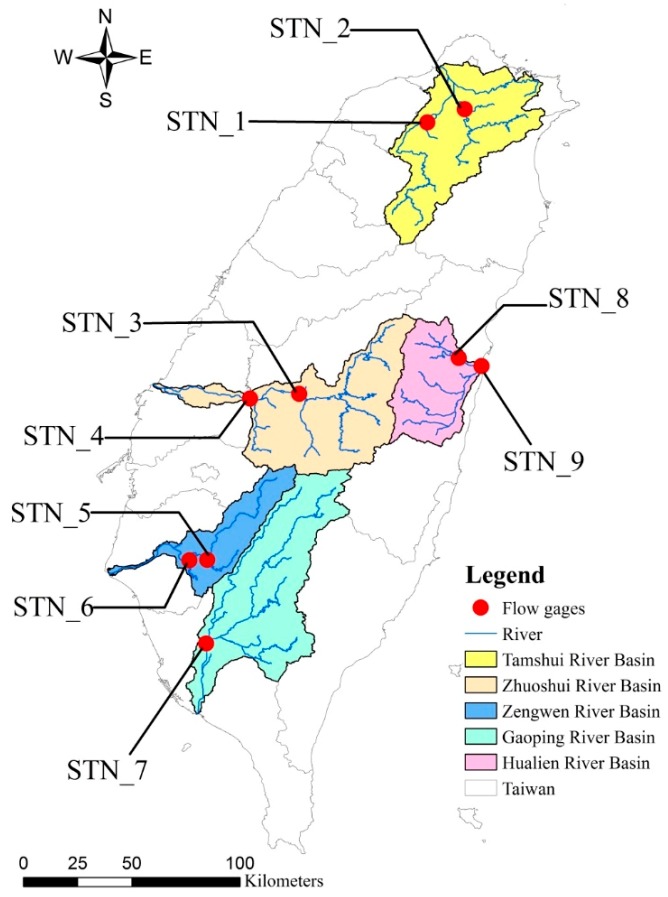
Locations of gauging stations in the five selected basins.

To investigate the spatiotemporal variation of sediment export from basins in Taiwan, five basins which are located in northern, central, southern, and eastern Taiwan, were selected as the study area, i.e., Tamshui River basin (northern Taiwan), Zhuoshui River basin (central Taiwan), Zengwen River basin and Gaoping River basin (southern Taiwan), and Hualien River basin (eastern Taiwan) (Figure 1). Loacted in northern Taiwan, Tamshui River is the third longest river in Taiwan. Within the basin, the population is nearly 8 million, accounting for 30% of Taiwan’s population. It is the political, economic, and cultural center of Taiwan. With all-year continuous and stable flow, Tamshui River is one of the few rivers with shipping functions in Taiwan. The average annual rainfall is 2966.1 mm with 1883.5 mm in the wet season (May–October) and 1082.6 mm in the dry season (November–April). Zhuoshui River, located in central Taiwan, is the longest river in Taiwan; the drainage area of 3156.90 km^2^ is the second largest river basin in Taiwan. Due to uneven distribution of rainfall, there is a significant seasonal difference in the streamflow. The average monthly discharge is about 300 m^3^/s. Flows are high from June to September, and drop to 30 m^3^/s during December and January [28], showing a great disparity between the wet and dry seasons. The annual average rainfall is 2459 mm, with the wet season from May to September and the dry season from October to April in the next year. The name of Zhuoshui in Mandarin means “turbid river,” as influenced by geology, landslides, typhoons and heavy rains, the stream contains large amoutns of sands and muds. The annual sediment transport from the Zhuoshui River basin reaches 63.87 megaton, accounting for 20% of the total amount of 322.76 megaton in the entire Taiwan.

Two basins located in southern Taiwan were selected: the Zengwen River basin and the Gaoping River basin. Zengwen River is 138.5 km long and drains an area of 1176.7 km^2^. The annual average rainfall is 2303.5 mm, with the wet season from May to September and the dry season from October to April in the next year. Gaoping River is the second longest river in Taiwan. The large amonts of discharge and sediment transport are found due to high precipitation and steep slope in the basin. The annual average rainfall is 3046 mm. The annual sediment transport from the Gaoping River basin reaches 35.61 megaton with the unit-area sediment transport of 10,934 tons per km^2^. The Hualien River basin, located in eastern Taiwan is agricultural-dominated. With steep upstream tributary slope, water flow is rapid and carries a lot of sediments when a typhoon flood occurrs. The annual average rainfall is 2100 mm, with the wet season from June to November and the dry season from December to May in the next year. The annual sediment transport in the Hualien River basin reaches 20.5 megaton. 

For those basins discussed above, hydrological and sediment data from 2000–2017 for a total of nine gauging stations at the upstream and/or downstream of a basin were collected from the Water Resources Agency, Ministry of Economic Affairs (http://gweb.wra.gov.tw/wrhygis/) for analysis. A total of nine gauging stations include: Sanhsia (STN_1), Hsiulung Bridge (STN_2), Yufeng Bridge (STN_3), Chunyun Bridge (STN_4), Yutien (STN_5), Erhchi Bridge (STN_6), Lilin Bridge (STN_7), Jenshou Bridge (STN_8), and Hualien Bridge (STN_9) (Figure 1 and Table 1). Among these stations, STN_7 receives the highest annual discharge. Table 2 and Table 3 show the descriptive statistics of measured continuous water discharge, and corresponding data on water discharge and sediment during 2000–2017 at the nine gauging stations, respectively. The corresponding data on water discharge and sediment concentration are sampled as instantenous data, and the sediment loading is calculated as sediment concentration multiplied by the corresponding water discahrge (Table 3). STN_7 located in the Gaoping River basin has the highest mean continuous water discharge of 222.72 m^3^/s (Table 2), while two stations (STN_3 and STN_4) in the Zhuoshui River basin have the highest sediment concentration (6713.15 mg/L and 5198.40 mg/L, respectively) and sediment load (612,717.64 ton/day and 746,673.83 ton/day, respectively) (Table 3). Obviously, sediment load was more affected by sediment concentration than by water discharge.

### 2.2. Selected Typhoon Information

**Figure 2:**
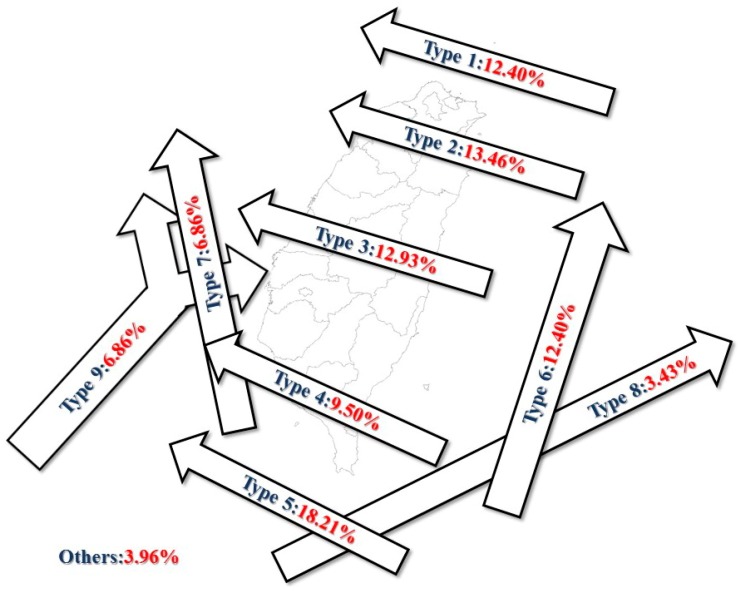
Types of typhoon paths and the percentage of occurrence (1911–2017) [29].

Based on the typhoon invasion path classification standard of the Central Weather Bureau in Taiwan, typhoon paths are categoraized into ten types (Figure 2). Among the ten paths, the major paths are type 1, 2, 3, 5, and 6, and the occurrence percentage of each major path type accounted for more than 10% of the total typhoon occurrence during 1911 to 2017. In Figure 2, typhoons with path types 2 and 3 invaded Taiwan from the east coast and across the island to the west through northen and central Taiwan, respectively. Typhoons with path types 1 and 5 went from southeast to northwest and passed the northern and southern sea areas of Taiwan, respectively, without making land on the island. Path type 6 typhoons passed by the eastern sea area of Taiwn from south to north along the east cost of the island. Generally, typhoon invasions increase the amount and intensity of rainfall in the affected areas. The Tamshui River basin located in northen Taiwan was more affected by typhoons with path types 1 and 2; the Zhuoshui River basin located in central Taiwan was affected by typhoons with type 3. Path type 3 and 4 typhoons affected the Zengwen and Gaoping River basins in southern Taiwan. The Hualien River basin located eastern Taiwan was affected by type 2 and 3 typhoons since those typhoons landed on Taiwan from the east coast near the river basin.

**Figure 3:**
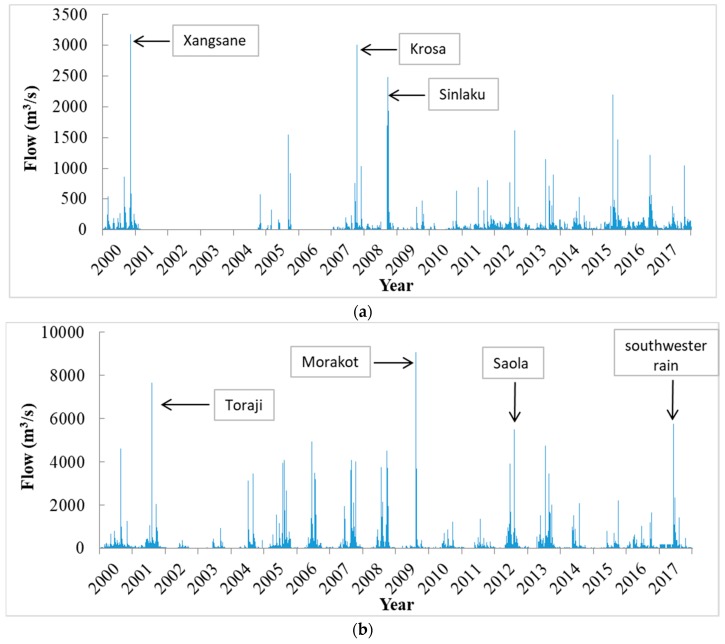

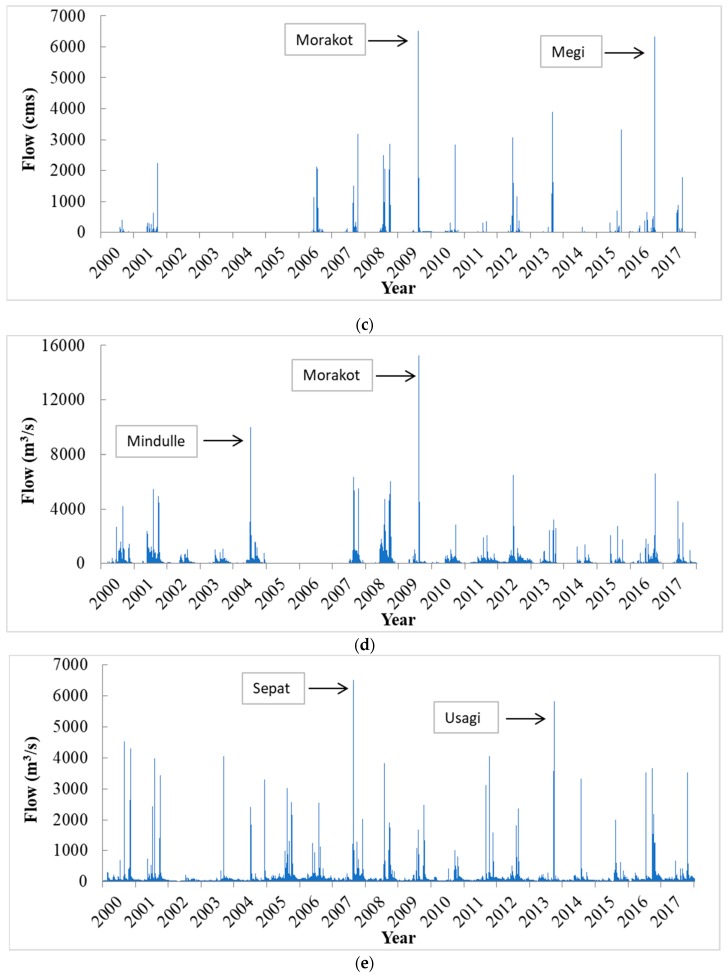
Historical daily streamflow of downstream gauging stations in the five river basins. (**a**) Tamshui River basin (STN_2); (**b**) Zhuoshui River Basin (STN_4); (**c**) Zengwen River Basin (STN_6); (**d**) Gaoping River Basin (STN_7); (**e**) Hualien River Basin (STN_9).

Figure 3 shows the daily streamflow during 2000–2017 at the downstream gauging stations of each river basin. The highest maximum daily streamflow during the period were identified with the corresponding typhoons, which are mostly path types 2 (typhoons Krosa, Sinlaku, Saola), 3 (typhoons Toraji, Morakot, Megi, Sepat), 5 (typhoon Usagi), and 6 (typhoons Xangsane, Mindulle) typhoons. Furthermore, a list of cumulated daily discharge and maximum sediment concentration during indentical typhoon events of 2000–2017 is shown in Table 4.

For STN_2 in the Tamshui River basin, typhoons Krosa and Sinlaku of path type 2, and typhoon Xangsane of path type 6 brought the top three greatest maximum daily streamflows and cumulated daily discharges (Figure 3a and Table 4a). However, the maximum sediment concentration from typhoons Krosa and Sinlaku was lower than that of typhoons Soudelor, Megi, Matmo, Fungwong (path type 3), and Xangsane (path type 6) (Table 4b). This shows that typhoon path could have some influence on how sediment will be eroded from the landscape. For STN_4 at the Zhuoshui River basin, several typhoons (Toraji, Haitang, Bilis, Sepat, Krosa, Sinlaku, Morako, Saola) brought very high cumulated daily discharges (>7000 m^3^/s) (Table 4a). Among these typhoons, typhoon Toraji, Morakot and Saola were identified for their highest maximum daily streamflow (Figure 3b). These typhoons belong to same path types (Type 2 and 3), showing the specific types of typhoon path would have a greater influence on the Zhuoshui River basin. Moreover, typhoons of path type 3, such as typhoon Sepat and Matmo would have greater power to flush the sediments and resulted in large numbers of maximum sediment concentration (Table 4b).

For STN_6 at the Zengwen River basin, the top two values of cumulated daily discahrge and maximum daily streamflow were found during typhoons Morakot and Megi (path type 3, Figure 3c and Table 4a). The maximum sediment concentration during typhoon Morakot (path type 3), Fanapi (path type 4), and Nari (path type 10) were obviously higher and between 6288–7278 ppm (Table 4b). However, the cumulated daily discharge during typhoons Fanapi and Nari were one-third and one-fourth of the discharge during typhoon Morakot, respectively (Table 4a). This shows that typhoons of path type 3 directly passed through the upstream of the Zengwen River basin could easily erode the sediments, and thus sediment concentration was not diluted due to high level of discharge.

For STN_7 at the Gaoping River basin, typhoon Morakot brought the highest cumulated daily discharge, followed by typhoons Mindulle and Sinlaku (Table 4a), and the heighest maximum daily streamflow during typhoons Morakot and Mindulle was found to be significantly larger than that in other events (Figure 3d). Moreover, typhoon Toraji, which belongs to path type 3 like typhoon Morakot, had a greater maximum sediment concentration than that of typhoon Sinkalu (path type 2), but the cumulated daily discharge in typhoon Toraji was smaller than that in typhoon Sinkalu, showing that typhoons of type 3 could have greater influence on sediment discharge than typhoons of type 2. For STN_9 at the Hualien River basin, typhoons Sepat and Usagi have the highest maximum daily streamflow and cumulated daily discharge (Figure 3e and Table 4a), and the highest maximum sediment concentration was found during typhoon Toraji (Table 3). Typhoons of path type 3 (typhoons Sepat abd Toraji) showed more impacts on both water discharge and sediment concentration in the Aualien River basin.

### 2.3. Sediment Rating Curves (SRC)

In order to quantify the characteristics of sediment discharge in different river basins, we collected corresponding data on water discharge and sediment from 2000 to 2017 at nine gauging stations throughout these basins. Sediment rating curve (SRC) is one of the most common methods applied in assessing sediment load in rivers, which relates the water flow to the amount of suspended sediment by using regression analysis and as a power equation [30,31]. Some studies defined SRC as the relationship between water discharge (Q, m^3^/s) and sediment load (Qs, ton/day or kg/s) [31,32,33], while some defined SRC as the relationship between water discharge (Q, m^3^/s) and suspended sediment concentration (Cs, mg/L) [6,7,8,12,13,14,15,34,35,36].

In this study, we first grouped sediment concentrations into five groups by using the exceedance probability (EP) of 10%, 25%, 50%, and 75%. Then, we applied a regression analysis to obtain a power function between the corresponding suspended sediment discharge (Qs) and water discharge (Q) for different groups to evaluate the how sediment concentration influence the relationships between sediment discharge and water discharge. The water discharge used to construct the sediment rating curve was chosen as the instant water discharge at which time the suspended sediment concentration was measured. In other words, the water discharge and the suspended sediment concentration taken at the same time were used for sediment rating curve construction.

In order to obtain the best performed statistical relationship between the suspended sediment concentration (Cs) and the water discharge (Q) for each watershed, two regression models (e.g., linear and polynomial) were established using transformed data (e.g., log and square root) [6,14]. The most commonly used sediment rating curve is a power function [13,34]:

(1) $$\mathrm{Cs}={\alpha Q}^{\beta }$$

where Cs is the suspended sediment concentration (mg/L); Q is water discharge (m^3^/s); α and β are regression coefficients [37]. The coefficient α can be regarded as an index of erosion severity in the river channel [38]. A high value of α indicates that the area is characterized with easily erodible materials and high loads of transported materials. The coefficient β depicts the erosive power of the river with a large β value indicates a small increase in discharge results in a strong increase in erosive power of the river. The β coefficient is related to channel morphology, the erodibility within the river basin, grain-size distribution of sediment, and regional climate variation, thus many studies have compared the values of the β coefficient of different rivers for evaluating the sediment transport characteristics in different basins [38,39,40].

Different statistical criteria were applied to evaluate the fitness of the regression models. The selected criteria include: correlation coefficient (r), standard error of estimate, and relative and absolute error of estimation [14]. The model with higher value of correlation coefficient and smaller value of other criteria was regarded as best fitted model [6,13]. The relative error of estimation is calculated as follows:

(2) $$\mathrm{RE}=\frac{Cs_{obs}-Cs_{est}}{Cs_{obs}}\;\times 100$$

where *Cs_obs_* and *Cs_est_* are observed and estimated sediment concentration (Cs), respectively. The positive RE indicates an underprediction of measured value, while the negative RE indicates an overprediction.

Since the suspended sediment concentration was not automatically monitored daily and the measurements were not continuous, the average daily mean suspended sediment concentration was estimated using the sediment rating curve and the daily mean discharge. Then the daily sediment load was obtained as the product of daily mean discharge and the daily mean suspended sediment concentration as shown in the following equation:

(3) Qs =Q×Cs×0.0864

where Q is measured water discharge (m^3^/s), Cs is the daily mean suspended sediment concentration (mg/L) from a rating curve, and Qs is estimated sediment load (ton/day). The daily sediment loads are then calculated for mean monthly sediment load (ton/day) and mean annual sediment load (ton/day). The possible error may result from the fluctuation sediment concentration and water discharge, intermittence of sediment concentration measurements, and the difference between the instant water discharge and the daily mean water discharge.

### 2.4. Flow Duration Curve

In order to differentiate the relationship between sediment concentration and water discharge at various sediment concentrations, we categorized the observed data by using flow duration curve (FDC). FDC is a cumulative distribution function that shows the percentage of specific discharge that is equaled to or exceeded [41]:

(4) $$P_{m}=P\left(Q>q_{m}\right)=\frac{m}{\left(n+1\right)}$$

where P_m_ is the exceedance probability (EP) associated with q_m_; q_m_ is the discharge ranked at the m^th^ place; *m* is the order from 1 (associate with the largest discharge) to *n* (associate with smallest discharge); *n* is total number of observed data. It should be noted that Q and q_m_ can be replaced by other variables, such as sediment concentration and sediment load. In this study, we applied flow duration curve (FDC) to identify different levels of sediment concentration, and then developed the relationships for each corresponding group of sediment load and water discharge. Moreover, the estimated daily sediment load and daily measured discharge of each subwatershed were analyzed and the exceedance probability (EP) of 10%, 25%, 50%, and 75% for sediment load and water discharge can be further identified.

## 3. Results

### 3.1. Relationship between Sediment Load and Water Discharge

**Figure 4:**
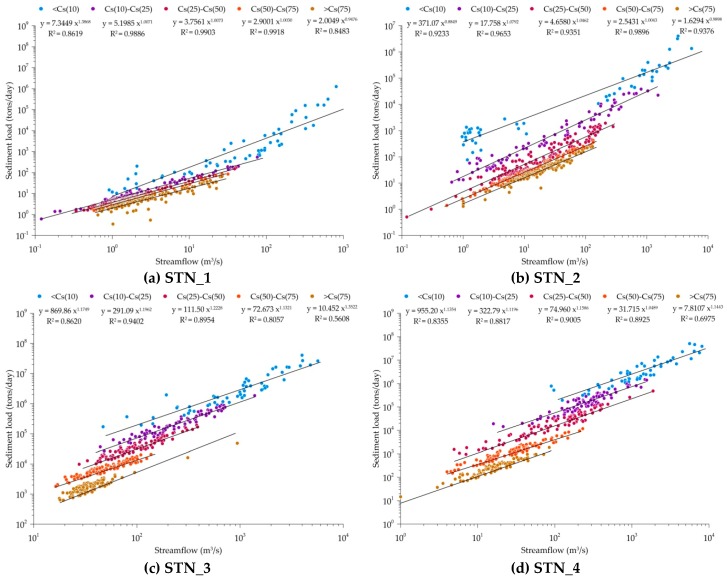

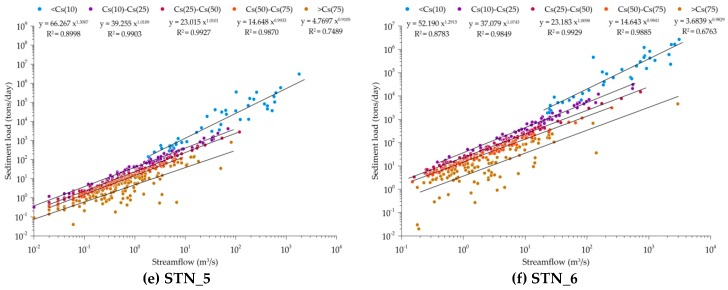

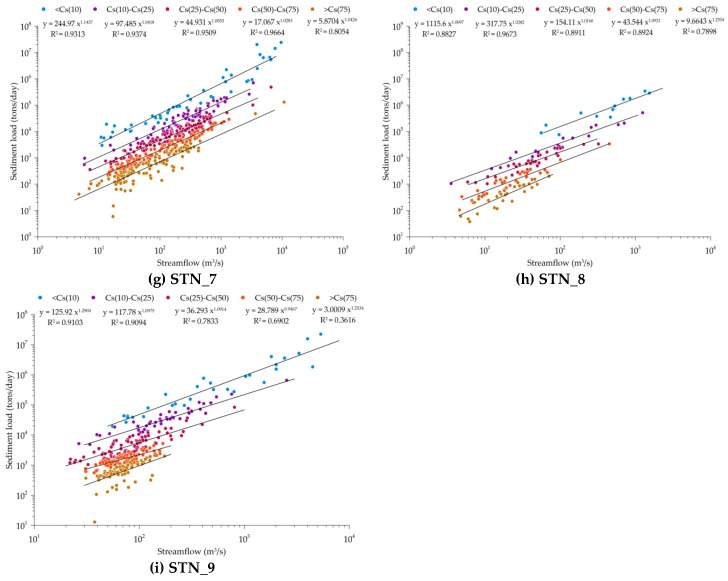
Sediment load and discharge relationships grouped by sediment load (ton/day).

Many studies have indicated the significant relationship between sediment and water discharge [9,10,11]. Especially, the typical steep hillslopes found in Taiwan result in high streamflow speed, and thus the sediment exports brought by heavy rainfalls can be significant. Studies have indicated that the channel morphology changed rapidly in Taiwan, and thus the sediment rating curves need to be updated for every year [42,43]. In order to further investigate the relationship between sediment load and discharge at different levels of sediment concentration, we applied the FDC to categorize the data into five groups by using different thresholds of sediment concentration. The values of C_s(10)_, C_s(25)_, C_s(50)_, and C_s(75)_ indicate the sediment concentration threshold that 10%, 25%, 50%, and 75% of the observed sediment concentration data was equal to or exceeded it, respectively. The statistical analysis of the relationship between sediment load and discharge of various exceedance groups is shown in Figure 4. In the figure, we investigated effect of sediment concentration level on the power of the relationship between the sediment load and discharge. It is found that the grouped relationships are generally better than the overall relationship. For example, the *R*^2^ values of the grouped relationship at STN_2 and STN_7 are all greater than 0.8, while the *R*^2^ values of the overall relationships are less than 0.7. This finding shows that different erodible materials could be identified by the sediment concentration, and thus a more significant relationship between sediment load and discharge could be defined.

Moreover, the relationship between sediment load and discharge was relative weak with the *R*^2^ values less than 0.7 for the low sediment concentration group (>C_s(75)_) at STN_3 (*R*^2^ = 0.5608), STN_4 (*R*^2^ = 0.6972), STN_6 (*R*^2^ = 0.6763), and STN_9 (*R*^2^ = 0.3616). The sediment concentration in the stream may vary with the intensity, frequency and amount of rainfall. The streamflow hydrograph follows the rainfall hydrograph, while the sediment concentration regime does not. The sediment concentration in the stream is usually higher after the rainfall event, and thus the sediment load of certain magnitude of discharge would be greater than the sediment load of same magnitude of discharge before rainfall. During the rainfall period, sediment is transported with the power of streamflow brought by the rainfall. Thus, we could see a relative good relationship between sediment load and discharge for the group of high sediment concentration when rainfall occurrs. Such relationship at different gauging stations also indicates the various responding characteristics of channel morphology and erosive materials to rainfall. Moreover, the relationships for the groups of C_s(10)_–C_s(25)_, C_s(25)_–C_s(50)_, and C_s(50)_–C_s(75)_ performed better than that of the other two distinct groups (<C_s(10)_ and >C_s(75)_), indicating a more stable relationship for sediment concentration ranging between C_s(10)_ and C_s(75)_.

Table 5 shows the thresholds of sediment concentration for the nine gauging stations. Sediment concentration was either higher at the downstream gauging station than that at the upstream gauging stations for the Tamshui River basin and the Zengwen River basin, or it was lower at the downstream gauging station for the Zhuoshui River basin and the Hualien River basin. It was caused by the steeper slope at the upstream drainage area; sediment flushed from the upstream was able to settle down and there were less erodible sediment materials at the downstream area. Among these five basins, the sediment concentration was the highest in the Zhuoshui River basin, followed by that in the Hualien River basin, Gaoping River basin, Zengwen River basin and Tamshui River basin. The distributions of sediment concentration of STN_5 and STN_6 were quite similar, indicating a stable channel morphology and consistent components of transported sediment in the Zengwen River basin. In the Hualien River basin, more than 50% of observed data at the upstream during 2000–2017 had sediment concentrations greater than 1104 ppm, while less than 25% of the data at the downstream had sediment concentrations greater than 1263.5 ppm. In the Tamshui River basin, the C_s(25)_ sediment concentration at the downstream gauging station was even higher than the C_s(10)_ sediment concentration at the upstream gauging station. Such various sediment concentration thresholds at different sections of the watershed could provide information to soil and water conservation manegers to implement management practices.

### 3.2. Sediment Rating Curves

The sediment data were collected two to five times a month, so the number of datapoints in a year was limited. Thus, we used the sediment rating curve to estimate the daily sediment concentraion based on the daily streamflow data. Although selection of statistical methods to fit the sediment rating curve could result in inaccuracies in predicted instantaneous suspended sediment concentration [13], several studies have been done to compare the SRC model performance of different watersheds [6,12,13,14]. Table 6 shows the perforamnce evaluation of sediment rating curves based on two statistical models (linear and second-order polynomial) using two types of transformed data (log and square root) of the nine gauging stations during 2000–2017. It was found that the SRC for most stations (STN_3, 4, 5, 6, 7, 8, 9) are generaly better fitted by linear regression than by second-order polynomial regressaion. Some stations (STN_2, 3, 4) with log transformed data showed better fitting performance, while some stations (STN_1, 5, 6, 7, 8, 9) performed better fitted model with square root transformed data. STN_1 fitted with polynomial regression using square root transformed data has the highest correlation coefficient (0.89) and the estimated sediment concentration range (39.70–12,302.12 mg/L) was similar to the range of measured sediment concentration (2.00–18,191.00 mg/L). However, located in the Tamshui River basin, the SRC of STN_2 showed different performance whereby polynomial regression using log transformed data was the best fitted model. The mean of estimated sediment concentration is smaller than the mean of measured one, which agrees with [13] who indicated that sediment load is likely underestimated when using log transformed data. However, the maximum estimated sediment concentration is three times greater than that of the measured one, indicating large underestimated low sediment concentration data and overestimation for high sediment concentrations. The difference in data transformation for STN_1 and STN_2 in the Tamshui River basin reflects the effect of different physical local characteristics, contribution of water discharge, and sediment availability. Both SRC of STN_3 and STN_4 at the Zhuoshui River basin performed consistently with any of the statistical models using different types of transformed data with very good correlation coefficient ranging between 0.81 and 0.84. When using log transformed data of STN_3, the mean and maximum estimated sediment concentrations (6361.50 mg/L and 156,546.63 mg/L, respectively) by the linear regression model were close to those of measured sediment concentration (6713.15 mg/L and 118,000.00 mg/L, respectively).

Based on the relationship between back-transformed estimated sediment concentration and original measured sediment concentration of STN_4, it was found that the relationship of sediment concentration estimated by linear regression model (*R*^2^ = 0.5018) is better than that of sediment concentration estimated by polynomial regression model (*R*^2^=0.4344). Thus, the best fitted model for STN_3 and STN_4 at the Zhuoshui River basin is the linear regression based on log transformed data.The relationship between sediment concentration and water discharge at the Zengwen River basin (STN_5 and 6), the Gaoping River basin (STN_7), and the Hualien River basin (STN_8 and 9) was better fitted by the linear regression using log transformed data, in terms of higher correlation coefficient and better estimated mean and maximum sediment concentrations. The value of slope coefficient shows how the sediment concentration is influenced by the increase of water discharge. The greater slope coefficient at the Hualien River basin indicate a small increase in discharge would result in a stronger increase in erosive power of the river, while the erosive power of the river at the Gaoping River basin would not be greatly affected by the increase in discharge. Moreover, both the upstream stations (STN_5 and STN_8) have greater slope coefficients than the downstream stations (STN_6 and STN_9). This finding will be useful to explain the source of sediment concentration at the downstream.

### 3.3. Flow Duration Curve

**Figure 5:**
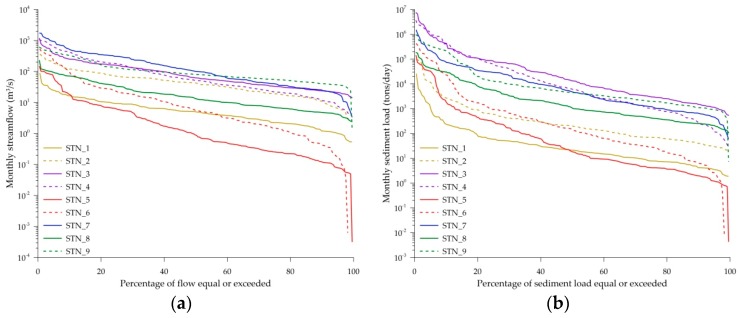
Monthly discharge and sediment export. (**a**) streamflow; (**b**) sediment load.

Figure 5 shows the flow duration curve (FDC) derived by using monthly discharge which is the average of daily streamflow in a month, and monthly-averaged daily sediment load which is the average of daily sediment load in a month calculated as the daily measured streamflow multiplied by the daily sediment concentration estimated by the best fitted regression model (Table 6). The FDC is then used to identify the percentage of specific discharge or sediment load that is equaled to or exceeded. In this study, we selected the thresholds of discharge and sediment load that was equaled to or exceeded 10%, 25%, 50%, and 75% of data during the entire period (Table 7). Q_10_, Q_25_, Q_50_, Q_75_ denote the threshold of monthly discharge that was equal to or exceeded 10%, 25%, 50%, and 75% of data, while Sed_10_, Sed_25_, Sed_50_, Sed_75_ denote the threshold of monthly sediment load that was equal to or exceeded 10%, 25%, 50%, and 75% of data.

**Figure 6:**
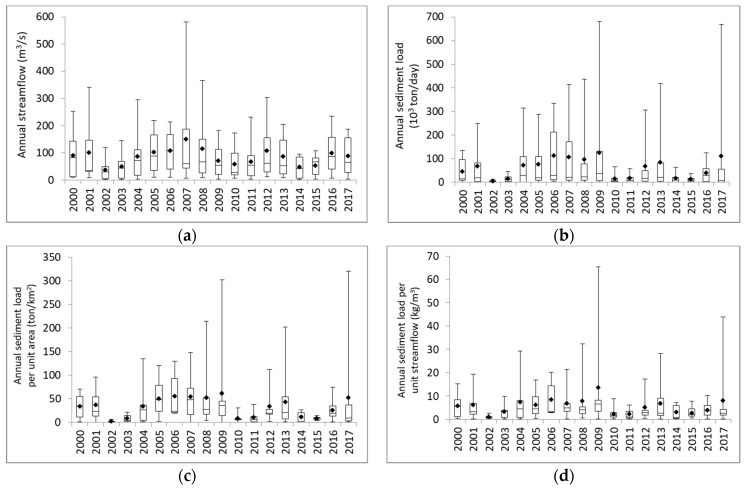
Temporal variation of annual streamflow and sediment load. (**a**) Annual streamflow; (**b**) Annual sediment load; (**c**) Unit-area sediment load; (**d**) Unit-discharge sediment load (Note: dots denote the average value of the data).

The steeper the slope of the flow duration curve is, the larger the difference between the normal discharge and peak discharge is. The FDC of the Tamshui River basin is the least steep, followed by that of the Hualien River basin in eastern Taiwan, and basins in central and southern Taiwan (Gaoping River basin, Zhuoshui River basin, and Zengwen River basin) (Figure 6a). The large difference between Q_10_ and Q_75_ discharge was found in the Zhuoshui River basin, Gaoping River basin and the downstream of the Hualien River basin, indicating seasonal variation of wet and dry seasons is more influential in southwestern and eastern Taiwan than in northern Taiwan. As the Gaoping River basin is the largest basin in Taiwan, the Q_10_, Q_25_, and Q_50_ discharge at STN_7 are the highest among all stations (Table 7). However, it is found that Q_75_ discharge at STN_7 is smaller than that at STN_9, indicating discharge at the Hualien River basin is mostly high among all stations. Moreover, the Q_10_ discharge at STN_4 at the downstream of the Zhuoshui River basin and STN_7 at the downstream of the Gaoping River basin are much greater than that at other stations, showing more soil and water conservation measures need to be implemented to prevent the damage during extreme events. 

Generally, the trend of sediment duration curves follow the flow duration curves (Figure 5). Thus, the sediment duration curve of the Tamshui River basin is the flattest, followed by that of basins in eastern and southwestern Taiwan. The Sed_10_ at the Zhuoshui River basin (348,324.65 ton/day and 409,247.80 for STN_3 and STN_4, respectively) and at the downstream of the Hualien River basin (207,677.94 ton/day for STN_9) are much greater than other stations. The large differences in Q_10_ and Q_75_ discharge (215.86 m^3^/s–388.1 m^3^/s) at STN_3, 4 and 9 resulted in differences in Sed_10_ and Sed_75_ ranging between 205686.04 ton/day and 408,230.62 ton/day. The results showed that an extremely large amount of sediment load should be expected when the monthly discharge exceeds the Q_10_ threshold. 

The spatial variation in the thresholds of sediment load and discharge is expected that the downstream gauging station would receive greater sediment load and discharge than the upstream stations. However, it is opposite in the Zhuoshui River basin for Q_50_, Q_75_, Sed_25_, Sed_50_ and Sed_75_. Moreover, the downstream (STN_4) Sed_75_ sediment load in the Zhuoshui River basin is smaller than the downstream (STN_9) Sed_75_ sediment load in the Hualien River basin, while the upstream (STN_3) Sed_75_ sediment load in the Zhuoshui River basin is much greater than the upstream (STN_8) Sed_75_ sediment load in the Hualien River basin. This indicates that the difference in channel morphology and landscape of the two river basins; and the soil in the upstream Zhuoshui River basin is more erodible than the downstream soil during the normal discharge conditions. As the eroded soil from the upstream is temporarily stored between upstream and downstream channels, the soil can be eroded again when the extreme discharge or typhoons occur. 

## 4. Discussion

### 4.1. Spatial and Temporal Variation of Sediment Transport Capability

Many studies have indicated that discharge is the key factor affecting the amount of sediment load in the rivers [44]. In order to compare the characteristics of sediment transport in different watersheds, we first calculated the annual streamflow which is calculated as the average of the daily streamflow in a year, and the annual sediment load which is annual-averaged daily sediment load which is the average of daily sediment load in a year calculated as the daily measured streamflow multiplied by the daily sediment concentration estimated by the best fitted regression model (Table 6). Then, the annual unit-area sediment load is calculated by dividing the annual sediment loads at each gauging station by its drainage area, and also the annual unit-discharge sediment load by dividing the annual sediment loads by its annual discharge. 

Figure 6 shows the temporal variation in annual streamflow, annual sediment load, unit-area sediment load and unit-discharge sediment load in the five river basins from 2000 to 2017. The length of the box represents the variation of streamflow and sediment load in Taiwan of the specific year. Due to fewer typhoons during 2002–2003, 2010–2011 and 2014–2015, the annual streamflow was lower than other years (Figure 6a). Consequently, the annual sediment load, unit-area sediment load and unit-discharge sediment load (Figure 6b,d) were also low in those years. In contrast, the high values of streamflow and sediment load were found in years that had significant typhoon events. For example, the top three highest annual streamflows were found in 2001, 2007 and 2008 (Figure 6a), while the top three highest sediment loads and unit-area sediment loads (Figure 6b,c) were found in 2008, 2009 and 2017. For unit-discharge sediment load, the top three highest values were found in 2008, 2009 and 2017. The significant typhoon events that brought high values of streamflow or/and sediment loads include typhoon Toraji and Nari in 2000, Sepat and Krosa in 2007, Fungwong, Sinlaku and Jangmi in 2008, Morakot in 2009, Saola in 2012, Soulik and Usagi in 2013, and Haitang in 2017. The average unit-area sediment load in Taiwan during 2000–2017 ranged between 1.80 and 61.11 ton/km^2^ with the maximum value of 313.78 ton/km^2^ at STN_3 in the Zhuoshui River basin in 2017, while the average unit-discharge sediment load ranged between 0.99 and 13.58 kg/m^3^ with the maximum value of 60.26 kg/m^3^ at STN_3 in the Zhuoshui River basin in 2009 due to the invasion of typhoon Morakot during which heavy rainfalls brought abundant sediments. The time lag between the peak flow and peak sediment shown in Figure 6a,b may be linked with the occurrence of hysteresis effect, when the suspended sediment concentration values were different during the rising and falling limb for the same discharge [45]. Therefore, if the instant discharge and suspended sediment concentration were measured during the rising limb of water discharge, the resultant sediment load may be overestimated. In contrast, the sediment load may be underestimated if the sediment rating curve was constructed using the instant discharge and suspended sediment concentration measured during the falling limb.

**Figure 7:**
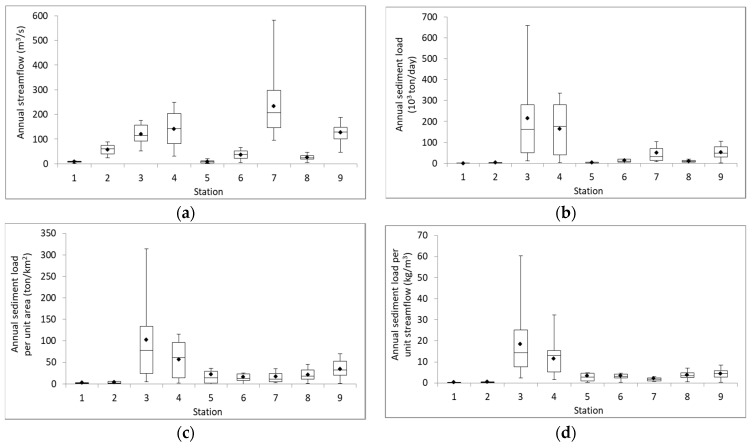
Spatial variation of annual streamflow and sediment load. (**a**) Annual streamflow; (**b**) Annual sediment load; (**c**) Unit-area sediment load; (**d**) Unit-flow sediment load (Note: dots denote the average value of the data).

In Figure 7, the spatial variation of sediment transport capacity in the five river basins is illustrated by the annual streamflow, annual sediment load, unit-area sediment load, and unit-discharge sediment load of the nine gauging stations. The downstream (STN_7) of the Gaoping River basin received the highest annual streamflow, while the Zhuoshui River basin received greater sediment loads than the Gaoping River basin (Figure 7a,b). It shows that even with large hydrological force the soil in the Gaoping River basin is much less erodible than that in the Zhuoshui River basin. It was found that the Zhuoshui River basin (STN_3 and STN_4) has greater annual sediment load, unit-area sediment load and unit-discharge sediment load than other basins, mainly due to the geological characteristics of the Zhuoshui River and its location to receive the impacts of landslides, typhoons, and extreme rainfalls that bring large amounts of sediments to the river.

Sediment transport capacity can spatially vary in a basin. In the Zhuoshui River basin, the sediment loads from the upstream (STN_3) was greater than the downstream (STN_4), in terms of the maximum and average values. Moreover, the high values of unit-area and unit-discharge sediment load at STN_3 and STN_4 also indicate a larger sediment transport capacity in per unit area and per unit flow of the Zhuoshui River basin than the capacity of other river basins. Thus, soil conservation should be effectively implemented in the upstream area. Typhoons are the main triggers to the significant abrupt sediment loads. The maximum unit-discharge sediment loads for each gauging station were 2.00 kg/m^3^ in 2001 for STN_1, 1.79 kg/m^3^ in 2008 for STN_2, 60.26 kg/m^3^ in 2009 for STN_3, 32.21 kg/m^3^ in 2009 for STN_4, 13.45 kg/m^3^ in 2000 for STN_5, 7.53 kg/m^3^ in 2009 for STN_6, 8.28 kg/m^3^ in 2009 for STN_7, 9.28 kg/m^3^ in 2014 for STN_8 and 8.97 kg/m^3^ in 2013 for STN_9. Typhoon Nari in 2001 and Typhoon Sinlaku and Jangmi in 2008 hit northern Taiwan and the flushed sediments led to the highest sediment load and unit-discharge sediment loads in STN_1 and STN_2 located in the Tamshui River basin, respectively. Moreover, STN_3 and 4 located in the Zhuoshui River basin, STN_6 in the downstream Zengwen River basin, and STN_7 located in the Gaoping River basin had the highest unit-discharge sediment load among all the stations in 2009 due to the huge amounts of sediments brought by typhoon Morakot.

### 4.2. Spatial Impact of Typhoon

**Figure 8:**
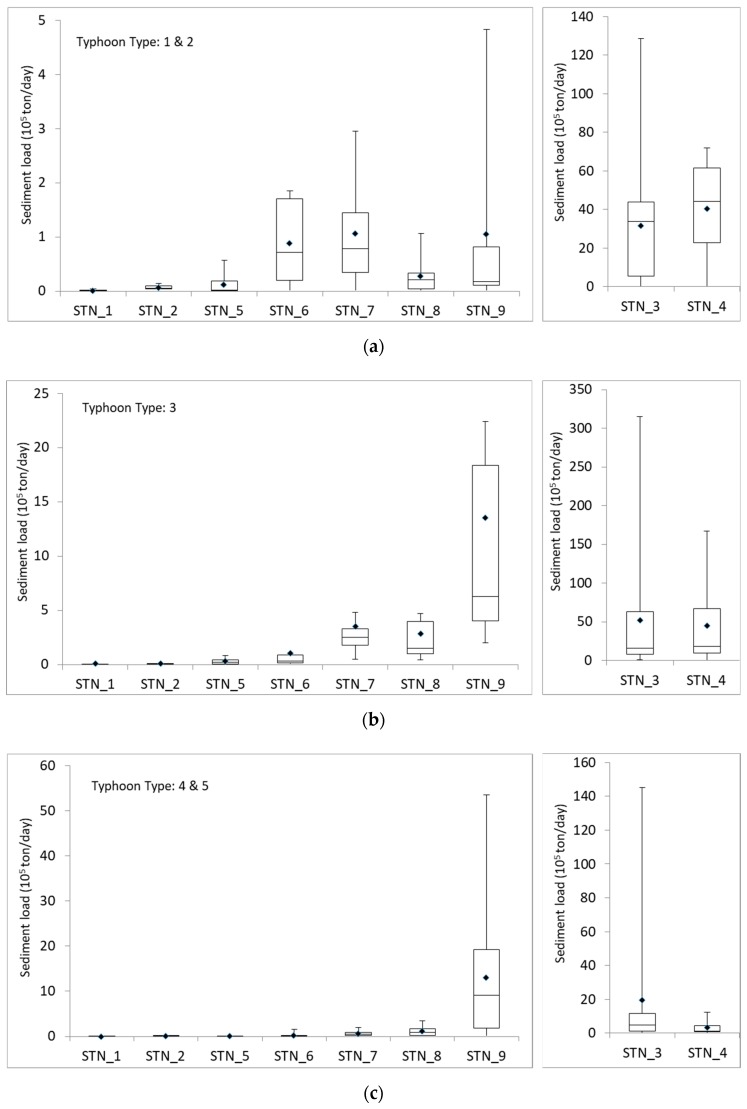

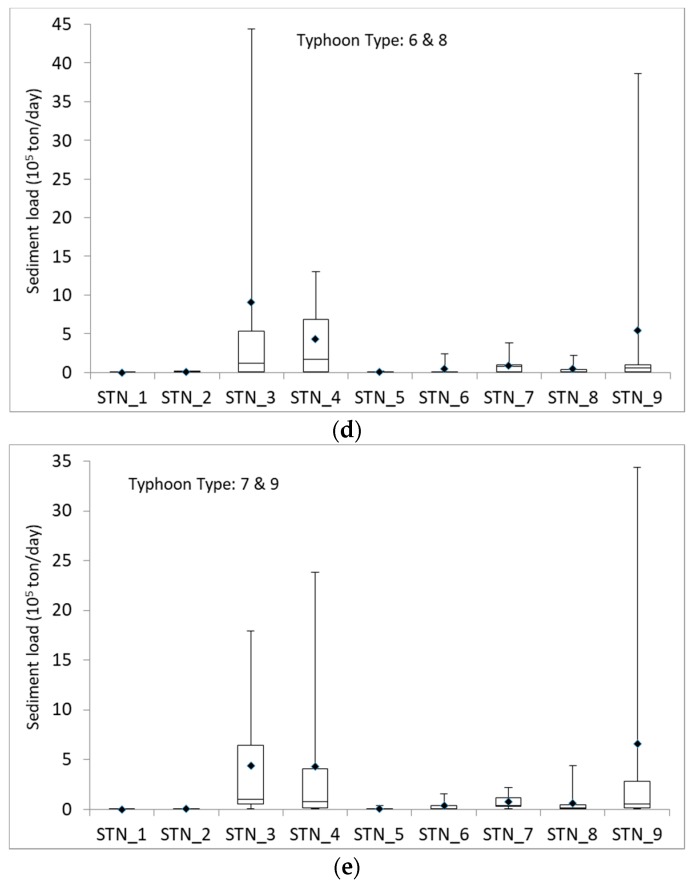
Average daily sediment load at nine gauging stations during typhoons of various types.

The invasion and travel paths of typhoons affect the sediment load and transport in different river basins. In Figure 8, we compared the sediment load recorded in nine gauging stations during typhoon events with different invasion path types, in order to show the spatial impacts of typhoon invasion path on river sediment load. Since the highest sediment loads recorded at STN_3 and STN_4 in the Zhuoshui River basin are generally larger than the values recorded in other stations up for one to two order magnitudes, we used another y-axis scale for the sediment load recorded in the two stations (STN_3 and STN_4).

In Figure 8a, typhoons of Type 1 and 2 brought the highest sediment loads in STN_3 and STN_4 located in the Zhuoshui River basin among all the typhoon events with different invasion paths. The average of daily sediment loads during typhoon event at STN_3 and STN_4 were greater than 3 × 10^6^ and 4 × 10^6^ tons/day, respectively, which is one order magnitude than the maximum daily sediment load at STN_9 in the Hualien River basin. Moreover, sediment loads at STN_6 and STN_7 were significantly larger during Type 1 and 2 typhoon events compared to the sediment loads brought by the other path types of typhoons. Type 1 and 2 typhoons landed on the east cost of northern Taiwan and preceded from east to west. Since this two path types of typhoons usually occur during summer and fall, and they usually trigger southwesterly flow when the typhoons leave Taiwan from the west coast. The accompanied effect of typhoon and southwesterly flow result in severe rainfall events especially in southern and central Taiwan, and thus high sediment loads in the Zhuoshui (STN_3 and STN_4), Zengwen (STN_6), and Gaoping (STN_7) River basin.

Typhoons of path Type 3, 4, and 5 invaded Taiwan from the east and two of the types (Type 3 and 4) landed Taiwan on the east coast. Therefore, the Hualien River basin (STN_9) located in eastern Taiwan had comparable sediment loads to those of the Zhuoshui River basin (STN_3 and STN_4) as shown in Figure 8b,c. However, the typhoon intensity was dampened when Type 3 typhoon events passed through Central Mountains to western Taiwan, and thus the sediment loads recorded at STN_3 and STN_4 during Type 3 typhoons were relatively low compared to those recorded during other events in the two stations (Figure 8b). Generally, the sediment loads in the river basins in western Taiwan were lower than those in eastern Taiwan during typhoons of Type 3, 4, and 5 (Figure 8b,c). For most of typhoon events, sediment loads in the Zhuoshui River basin (STN_3 and STN_4) were generally higher than other basins. However, the difference was smaller during the typhoon events of Type 6 & 8 and Type 7 & 9, which invaded Taiwan from the south towards northeast or northwest and usually did not land the island (Figure 8d,e). 

In sum, the top three highest sediment loads were recorded at STN_3, STN_4 and STN_9 for all path types of typhoons invaded Taiwan, suggesting that soil and water conservation is a key issue especially in the Zhuoshui and Hualien River basin. To prevent huge amounts of sediments from being flushed down to downstream areas and threaten the life and property of people, more focus should be put to these two river basins when implementing regulations preventing over-development and strategies of soil/water conserving measurements and facilities.

## 5. Conclusions

In this study, we selected five river basins (Tamshui River basin, Zhuoshui River basin, Zengwen River basin, Gaoping River basin, and Hualien River basin) to represent the hydrogeographic characteristics of northern, central, southern, and eastern Taiwan. Data from a total of nine gauging stations were analyzed for the spatiotemporal variation in streamflow and sediment load by using sediment rating curve (SRC) and flow duration curve (FDC). The relationships between water discharge and sediment loads of various levels of sediment concentration showed a very good correlation. By evaluating two types of SRC models (linear and polynomial regression) with two types of transformed data (log and square root), the best fitted SRC models with correlation coefficient greater than 0.5 were selected for each gauging station to estimate daily sediment concentration. 

Based on the results of flow duration curves, several thresholds of exceedance probability that streamflow or sediment load is equaled or exceeded were identified based on the observed data from 2000 to 2017. The variation among different river basins showed greater variation in the southwestern part of Taiwan. In the Zhuoshui River basin, the soil in the upstream is more erodible than the downstream soil during the normal discharge conditions. As the eroded soil from the upstream is temporarily stored between upstream and downstream channels, the soil is eroded again when the extreme discharge or typhoons occur. The mechanism of how sediment load was generated at different level of streamflow could be found by analyzing the flow duration curves. The thresholds of monthly streamflow and monthly-averaged daily sediment load can be useful for developing indicators for watershed management.

Among these five river basins, the Zhuoshui River basin generated the highest amount of sediment loads. Besides the Tamshui River basin, we found that sediment loads were more affected by sediment concentration than streamflow in other parts of Taiwan. Especially in southern and eastern Taiwan, sediment concentration was extremely high due to uneven distribution of rainfall seasons, steep slope and fragile geographical characteristics. Thus, unit-discharge sediment load and unit-area sediment load were the highest in the Zhuoshui River basin. 

From the analysis of spatial and temporal variation of sediment transport capability, occurrence of significant typhoon events directly affected the streamflow and sediment loads in the river basins. For example, Typhoon Morakot in 2009 brought the highest unit-discharge sediment load in the Zhuoshui River basin, downstream Zengwen River basin and Gaoping River basin. Moreover, spatial impact of typhoon on river basins was illustrated by the sediment loads recorded in the nine gauging stations during different typhoon path type events. Although different invasion paths of typhoons had an impact on sediment loads in different river basins, the Zhuoshui and Hualien River basins were usually impacted by typhoon events no matter what path type of typhoons invaded Taiwan. 

In sum, sediment concentration, the characteristics of watershed soil property and typhoons were the key factors for sediment loads. The results illustrate the spatiotemporal variations from the rating curves built for the five basins, which represent the diverse geomorphologic conditions in Taiwan. Implication of this study could provide an insight of basin ecosystem resistance to natural disturbance, and suggest further effectively implementing best management practices to prevent soil erosion. It is suggested that soil and water conservation is a key issue especially in the Zhuoshui and Hualien River basin, and more focus should be put to them when implementing regulations preventing over-development and strategies of soil/water conserving measurements and facilities.

## Figures and Tables

**Table 1:** General characteristics of the gauging stations.

Basin	Main Stream Length (km)	Basin Area (km^2^)	Upstream/Downstream Station	Station Name	Station Code	Drainage Area (km^2^)
Tamshui River basin (TSB)	158.7	2726.00	Upstream	Sanhsia	STN_1	125.34
			Downstream	Hsiulung Bridge	STN_2	750.76
Zhuoshui River basin (ZSB)	186.6	3156.90	Upstream	Yufeng Bridge	STN_3	2098.94
			Downstream	Chunyun Bridge	STN_4	2906.32
Zengwen River basin (ZWB)	138.5	1176.64	Upstream	Yutien	STN_5	160.53
			Downstream	Erhchi Bridge	STN_6	825.05
Gaoping River basin (GPB)	171.0	3256.85	Downstream	Lilin Bridge	STN_7	2894.79
Hualien River basin (HLB)	57.3	1507.09	Upstream	Jenshou Bridge	STN_8	425.92
			Downstream	Hualien Bridge	STN_9	1506.0

Source: Hydrological Year Book of Taiwan.

**Table 2:** Descriptive statistics of continuous water discharge for the five watersheds during 2000–2017.

	Station	STN_1	STN_2	STN_3	STN_4	STN_5	STN_6	STN_7	STN_8	STN_9
Statistics										
Size of dataset		4854	4375	6573	6571	6565	4866	5628	6575	6575
Mean (m^3^/s)		7.79	58.86	120.21	141.64	8.52	35.91	222.72	26.30	127.17
Median (m^3^/s)		2.20	27.56	54.25	40.00	0.55	3.91	80.59	10.18	70.00
Standard deviation (m^3^/s)		24.23	133.83	273.91	376.42	58.98	230.24	559.71	66.29	281.87
Maximum (m^3^/s)		688.00	3180.00	7884.90	9074.17	2190.00	6515.64	15,251.66	1997.24	6511.02
Minimum (m^3^/s)		0.02	0.01	1.80	0.32	0.01	0.01	2.23	0.02	0.01

**Table 3:** Descriptive statistics of corresponding data on water discharge and sediment for the five watersheds during 2000–2017.

	Station	STN_1	STN_2	STN_3	STN_4	STN_5	STN_6	STN_7	STN_8	STN_9
Statistics										
Size of dataset		577 (566) ^1^	525 (521) ^1^	551	519	523 (458) ^1^	399 (366) ^1^	466 (465) ^1^	430 (127)^1^	475 (308) ^1^
Data period		2000–2004, 2006–2017	2000–2004, 2006–2017	2000–2017	2000–2017	2000–2017	2000–2001, 2006–2017	2000–2004, 2007–2017	2000–2017	2000–2017
Water Discharge (m^3^/s)										
Mean		15.37	109.74	226.72	291.52	20.31	89.14	386.96	115.21	212.53
Median		1.71	14.57	60.47	44.51	0.77	4.25	131.00	33.70	78.93
SD ^2^		64.19	406.16	555.91	851.95	113.04	359.64	1054.45	248.37	564.24
Maximum		806.71	5400.00	5666.22	8094.29	1804.75	3094.93	10,798.00	1540.00	5350.00
Minimum		0.12	0.12	15.10	1.00	0.01	0.15	4.71	3.55	21.86
Sediment Concentration (mg/L)										
Mean		141.61	484.1	6713.15	5198.40	626.19	688.05	1312.47	3413.76	1761.38
Median		38.00	37.00	2180.00	795.00	211.50	214.50	335.00	1104.00	373.00
SD		897.66	1664.45	13,280.19	12,160.51	2446.39	2626.05	3957.76	6372.63	4798.51
Maximum		18,191.00	14,392.00	118,000.00	105,500.00	39,642.00	41,926.00	60,010.00	30,942.00	48,600.00
Minimum		2.00	4.00	18.00	10.00	1.00	1.00	4.00	69.00	4.00
Sediment Load (ton/day)										
Mean		4224.72	25,856.90	612,717.64	746,673.83	12,772.56	34,191.13	226,077.09	118,194.74	214,255.76
Median		5.53	51.12	11,554.10	2429.57	12.23	59.21	3511.35	2804.04	2284.04
SD		56,339.58	242,727.83	3,046,214.73	4,088,046.60	148,755.62	208,199.63	1,723,341.90	461,253.68	1,621,744.82
Maximum		1,267,912.76	4,046,386.74	40,558,259.17	51,102,235.84	3,062,159.50	2,682,041.58	24,193,963.01	3,438,547.20	22,464,864.00
Minimum		0.35	0.51	38.26	10.11	0.04	0.02	5.88	37.51	12.99

^1^ Non-continuous observed data during 2000–2017, exclude zero value; ^2^ Standard deviation.

**Table:** (a) Accumulated daily discharge (m^3^/s-day).

Period	Type	Typhoon	STN_1	STN_2	STN_3	STN_4	STN_5	STN_6	STN_7	STN_8	STN_9
**2000/08/21–08/23**	3	Bilis	109.64	1101.80	3081.60	4860.00	2197.75	415.60	4514.00	1375.92	4759.40
**2000/10/30–11/01**	6	Xangsane	386.69	3593.60	2247.00	1495.30	8.03	35.55	2791.00	818.70	7042.00
**2001/07/28–07/31**	3	Toraji	0.91	*	5589.60	9925.00	154.54	814.70	8651.00	1482.90	4638.40
**2001/09/13–09/19**	10	Nari	1539.13	*	1309.30	4881.00	991.74	3008.10	10,107.00	252.36	1633.80
**2004/06/28–07/03**	6	Mindulle	63.07	*	4751.44	3936.75	167.83	*	14,072.46	1699.19	5097.39
**2004/08/23–08/26**	1	Aere	806.70	*	3634.50	6326.06	34.13	*	3696.88	293.80	534.78
**2005/07/16–07/20**	3	Haitang	*	*	4145.65	8030.01	2221.44	*	*	1205.66	2763.59
**2006/07/12–07/15**	2	Bilis	*	*	1469.22	7775.90	1337.41	5084.88	*	166.03	631.72
**2007/08/16–08/19**	3	Sepat	102.04	182.77	6032.89	8733.49	966.18	1478.98	9978.37	2400.84	9260.34
**2007/10/04–10/07**	2	Krosa	*	4850.05	3791.27	7344.37	971.39	4522.31	8235.42	357.37	1809.76
**2008/07/26–07/29**	3	Fung–wong	185.35	*	2631.85	4272.75	831.36	3380.82	7558.38	1109.11	5506.57
**2008/09/11–09/16**	2	Sinlaku	583.71	6080.88	9739.47	11,820.72	1268.44	4340.33	13,840.91	611.55	4203.77
**2008/09/26–09/29**	2	Jangmi	404.19	3365.95	4141.88	5464.31	792.72	3980.17	7629.02	562.18	2706.49
**2009/08/05–08/10**	3	Morakot	*	1014.08	14,505.01	17,470.13	2412.07	11,967.59	36,012.07	1219.87	4145.62
**2010/09/17–09/20**	4	Fanapi	104.18	229.37	2371.08	2422.71	617.11	4135.96	4998.52	275.76	1803.28
**2011/08/27–08/31**	4	Nanmadol	41.24	631.18	1741.27	1142.41	121.36	487.81	5476.72	1376.61	5738.81
**2012/07/30–08/03**	2	Saola	647.43	2750.83	5159.94	8314.33	148.56	1948.87	2558.54	1190.46	4197.03
**2013/07/11–07/13**	2	Soulik	125.76	1306.44	3053.12	5195.44	124.67	183.99	2683.19	221.88	396.17
**2013/09/19–09/22**	5	Usagi	48.67	778.98	6300.24	3127.29	47.79	19.97	3352.63	863.60	9988.73
**2014/07/21–07/23**	3	Matmo	112.92	658.69	1276.41	2152.00	39.28	194.00	*	2108.49	3520.74
**2015/08/06–08/09**	3	Soudelor	619.02	3288.99	609.70	1199.55	377.52	1406.88	5275.49	527.46	2761.88
**2016/09/25–09/28**	3	Megi	362.16	2574.42	3665.09	2893.88	1112.28	8333.61	9747.07	700.95	3141.04
**2017/07/29–07/31**	7	Haitang	30.89	299.67	1778.22	2511.49	271.76	2538.97	4093.22	164.76	808.98

**Table:** (b) Maximum sediment concentration (mg/L).

Period	Type	Typhoon	STN_1	STN_2	STN_3	STN_4	STN_5	STN_6	STN_7	STN_8	STN_9
**2000/08/21–08/23**	3	Bilis	*	1886	*	*	*	*	91	21,600	3640
**2000/10/30–11/01**	6	Xangsane	24	2935	*	*	*	42	10,728	*	12,500
**2001/07/28–07/31**	3	Toraji	*	*	*	*	*	*	29,048	28,300	48,600
**2001/09/13–09/19**	10	Nari	5812	*	15,700	19,500	273	6288	9474	9140	13,000
**2004/06/28–07/03**	6	Mindulle	*	23	37,100	24,500	376	*	*	24,777	8944
**2004/08/23–08/26**	1	Aere	*	*	66,610	18,500	4120	*	*	*	*
**2005/07/16–07/20**	3	Haitang	*	*	25,600	22,800	4075	*	*	1120	5500
**2006/07/12–07/15**	2	Bilis	*	68	*	*	*	1181	*	*	*
**2007/08/16–08/19**	3	Sepat	*	1036	118,000	105,500	1771	*	320	*	*
**2007/10/04–10/07**	2	Krosa	*	451	*	*	*	3263	347	*	*
**2008/07/26–07/29**	3	Fung-wong	447	1770	*	*	1228	*	*	*	*
**2008/08/19–08/21**	2	Sinlaku	154	1622	53,500	2960	682	254	15,069	*	*
**2008/09/11–09/16**	2	Jangmi	504	752	*	*	*	*	12,100	*	*
**2009/08/05–08/10**	3	Morakot	165	1292	1620	33,300	19,638	7278	60,010	864	1203
**2010/09/17–09/20**	4	Fanapi	154	1061	80,700	54,200	834	7164	4534	*	4096
**2011/08/27–08/31**	4	Nanmadol	*	447	37,440	46,600	9779	143	*	*	*
**2012/07/30–08/03**	2	Saola	739	1014	23,500	55,600	*	97	1053	*	3016
**2013/07/11–07/13**	2	Soulik	3106	1794	54,500	31,950	7046	*	320	*	*
**2013/09/19–09/22**	5	Usagi	*	*	*	*	*	*	*	*	*
**2014/07/21–07/23**	3	Matmo	*	2481	56,880	73,340	515	4704	*	4774	4723
**2015/08/06–08/09**	3	Soudelor	18,191	14,392	21,480	15,530	7425	*	732	2894	2933
**2016/09/25–09/28**	3	Megi	4302	6194	*	*	2051	*	*	*	*
**2017/07/29–07/31**	7	Haitang	*	99	*	*	*	1431	*	1885	649

* denotes no observed data during the typhoon event.

**Table 5:** Thresholds of sediment concentration (mg/L) for gauging stations.

	Tamshui River Basin		Zhuoshui River Basin		Zengwen River Basin		Gaoping River Basin	Hualien River Basin	
	STN_1	STN_2	STN_3	STN_4	STN_5	STN_6	STN_7	STN_8	STN_9
C_s(10)_ (mg/L)	89.5	670	15,800	13,906	672.9	867.5	2477.8	6972	3828.3
C_s(25)_ (mg/L)	51	137	6065	4020	350.5	356	872	2947	1263.5
C_s(50)_ (mg/L)	38	37	2180	795	211.5	214.5	335	1104	373
C_s(75)_ (mg/L)	30	24	855	259	131	125	151	409.5	174

**Table 6:** Details and performance evaluation of sediment rating curve based on log transformation and square root transformation of the data in nine different subwatersheds.

Station	SRC	*R*	Std. Error of Estimation	Error (%)		Estimated Cs (mg/L)		
				Relative	Absolute	Mean	Maximum	Minimum
Linear Regression based on Log Transformed Data								
STN_1	y = 0.3258x + 1.5069	0.52	0.32	−3.71	14.13	50.50	284.39	16.10
STN_2	y = 0.1457x + 1.657	0.16	0.67	−10.51	27.62	71.28	158.79	33.33
STN_3	(y = 0.9925x + 1.4695) ^1^	0.83	0.35	−1.34	8.42	6361.50	15,6546.63	436.15
STN_4	(y = 0.8902x + 1.4403) ^1^	0.82	0.44	−2.51	12.47	3710.29	83,054.03	27.56
STN_5	y = 0.2639x + 2.3383	0.47	0.46	−5.65	16.90	366.26	1576.41	64.64
STN_6	y = 0.3442x + 2.0752	0.53	0.51	−10.09	23.30	397.93	1890.96	61.89
STN_7	y = 0.3832x + 1.8004	0.39	0.56	−5.12	17.89	847.75	2218.08	114.37
STN_8	y = 0.6991x + 1.9785	0.66	0.45	−0.75	12.12	1650.45	12,920.23	222.16
STN_9	y = 1.0096x + 0.6857	0.64	0.49	−4.04	15.62	664.47	28,173.84	109.20
Second-Order Polynomial Regression based on Log Transformed Data								
STN_1	y = 0.3937x^2^ − 0.386x + 1.5923	0.75	0.25	−2.63	10.96	88.23	6261.54	31.46
STN_2	(y = 0.5096x^2^ − 1.3376x + 2.4362) ^1^	0.63	0.53	−6.84	20.68	276.01	34,903.34	36.18
STN_3	y = −0.2138x^2^ + 1.942x + 0.4911	0.83	0.34	−1.25	8.26	5367.67	58,634.07	304.43
STN_4	y = 0.0891x^2^ + 0.5361x + 1.7427	0.83	0.44	−2.55	12.32	4665.51	158,043.15	55.30
STN_5	y = 0.1277x^2^ + 0.1835x + 2.2309	0.56	0.43	−5.24	16.04	264.68	15,227.04	146.21
STN_6	y = 0.0961x^2^ + 0.1334x + 2.0964	0.56	0.50	−10.01	22.72	335.96	5407.51	112.25
STN_7	y = 0.236x^2^ − 0.6574x + 2.8566	0.44	0.54	−5.02	17.71	755.51	11,073.75	250.49
STN_8	y = 0.0851x^2^ + 0.3901x + 2.2269	0.67	0.45	−2.29	12.16	1222.76	21,626.81	293.30
STN_9	y = 0.1426x^2^ + 0.3354x + 1.4409	0.64	0.48	−4.06	15.56	474.93	47,164.86	140.00
Linear Regression based on Square Root Transformed Data								
STN_1	y = 2.2589x + 2.5627	0.79	5.45	−9.19	36.27	111.92	4451.75	11.19
STN_2	y = 0.8034x + 7.615	0.37	16.63	−72.06	90.54	207.56	4442.57	62.30
STN_3	y = 4.399x + 13.407	0.84	28.41	−24.52	41.35	5905.85	118,706.87	930.31
STN_4	y = 3.3577x + 11.28	0.81	30.34	−39.56	58.33	4278.10	98,198.54	214.26
STN_5	(y = 2.8751x + 12.279) ^1^	0.67	12.81	−33.10	51.08	462.08	18,068.74	157.92
STN_6	(y = 1.4097x + 12.138)^1^	0.63	14.28	−49.13	67.22	484.13	8201.58	160.88
STN_7	(y = 0.9517x + 12.442) ^1^	0.51	21.67	−47.23	69.94	847.69	12,395.80	210.47
STN_8	(y = 3.5561x + 16.688)^1^	0.68	26.93	−32.54	53.98	2688.76	24,410.76	547.01
STN_9	(y = 2.4622x + 1.8633) ^1^	0.76	19.08	−37.97	59.70	1397.26	33,108.61	178.90
Second-Order Polynomial Regression based on Square Root Transformed Data								
STN_1	(y = 0.1464x^2^ − 0.4893x + 6.71) ^1^	0.89	4.08	−8.90	21.04	124.95	12,302.12	39.70
STN_2	y = 0.0233x^2^ − 0.2438x + 11.793	0.43	16.18	−72.34	90.57	221.97	14,327.48	124.44
STN_3	y = −0.0432x^2^ + 6.6677x − 2.5587	0.85	27.14	−18.92	36.72	5973.57	64,804.38	515.24
STN_4	y = −0.0287x^2^ + 5.1249x − 0.5083	0.84	28.75	−26.83	48.98	4373.83	52,104.44	21.05
STN_5	y = −0.0327x^2^ + 3.7052x + 11.258	0.68	12.71	−32.41	51.73	464.75	12,022.74	135.21
STN_6	y = −0.0314x^2^ + 2.6944x + 8.9414	0.67	13.72	−43.41	65.77	499.77	4414.39	99.60
STN_7	y = 0.0039x^2^ + 0.665x + 15.061	0.51	21.62	−48.48	70.88	848.33	15,945.53	273.00
STN_8	y = −0.0488x^2^ + 5.1768x + 9.2904	0.69	26.69	−30.26	51.86	2701.20	18,848.74	356.11
STN_9	y = −0.0097x^2^ + 3.0427x − 2.7374	0.76	19.01	−36.38	58.63	1400.70	28,197.77	127.16

^1^ The best fitted model.

**Table 7:** Flow duration curve analysis at nine gauging stations.

	Tamshui River Basin		Zhuoshui River $Basin		Zengwen River Basin		Gaoping River Basin	Hualien River $Basin	
	STN_1	STN_2	STN_3	STN_4	STN_5	STN_6	STN_7	STN_8	STN_9
Q_10_ (m^3^/s)	16.05	119.77	249.42	410.82	17.18	80.07	492.70	70.88	318.74
Q_25_ (m^3^/s)	9.28	68.58	157.07	178.98	6.03	21.76	305.32	33.05	130.17
Q_50_ (m^3^/s)	4.99	42.67	62.17	51.00	0.89	5.62	100.15	13.34	79.94
Q_75_ (m^3^/s)	2.25	19.25	33.57	22.01	0.26	1.48	40.45	6.91	55.42
Sed_10_ (ton/day)	227.28	2634.81	348,324.65	409,247.80	1638.54	21,689.22	71,961.33	29,170.00	207,677.94
Sed_25_ (ton/day)	58.60	585.27	78,884.05	61,471.48	290.23	944.51	29,084.70	5559.07	12,745.43
Sed_50_ (ton/day)	21.25	202.80	13,246.79	6140.17	18.38	142.20	4749.24	1064.93	4539.89
Sed_75_ (ton/day)	7.84	70.03	3052.09	1017.17	4.24	28.01	1216.87	424.97	1991.90
